# Chronic oral exposure to field-realistic pesticide combinations via pollen and nectar: effects on feeding and thermal performance in a solitary bee

**DOI:** 10.1038/s41598-019-50255-4

**Published:** 2019-09-24

**Authors:** Celeste Azpiazu, Jordi Bosch, Elisa Viñuela, Piotr Medrzycki, Dariusz Teper, Fabio Sgolastra

**Affiliations:** 1 Unidad de Protección de Cultivos, Escuela Técnica Superior de Ingeniería Agronómica, Alimentaria y de Biosistemas, Universidad Politécnica de Madrid (ETSIAAB-UPM), Av. Puerta de Hierro 2, 28040 Madrid, Spain; 2 CREAF, Universitat Autònoma de Barcelona, Cerdanyola del Vallès, 08193 Barcelona, Spain; 3 CREA-Consiglio per la Ricerca in Agricoltura e l’Analisi dell’Economia Agraria, Centro di Ricerca Agricoltura ed Ambiente, Via di Saliceto 80, 40128 Bologna, Italy; 4 Research Institute of Horticulture, Apiculture Division, 2 Kazmierska st., 24100 Puławy, Poland; 5 Dipartimento di Scienze e Tecnologie Agro-Alimentari, Alma Mater Studiorum Università di Bologna, viale Fanin 42, 40127 Bologna, Italy

**Keywords:** Entomology, Ecosystem services

## Abstract

Pesticide use is one of the main causes of pollinator declines in agricultural ecosystems. Traditionally, most laboratory studies on bee ecotoxicology test acute exposure to single compounds. However, under field conditions, bees are often chronically exposed to a variety of chemicals, with potential synergistic effects. We studied the effects of field-realistic concentrations of three pesticides measured in pollen and nectar of commercial melon fields on the solitary bee *Osmia bicornis* L. We orally exposed females of this species throughout their life span to 8 treatments combining two neonicotinoid insecticides (acetamiprid, imidacloprid) and a triazole fungicide (myclobutanil) via pollen and sugar syrup. We measured pollen and syrup consumption, longevity, ovary maturation and thermogenesis. Although bees consumed larger amounts of syrup than pollen, pesticide intake via syrup and pollen were similar. At the tested concentrations, no synergistic effects emerged, and we found no effects on longevity and ovary maturation. However, all treatments containing imidacloprid resulted in suppressed syrup consumption and drastic decreases in thoracic temperature and bee activity. Our results have important implications for pesticide regulation. If we had measured only lethal effects we would have wrongly concluded that the pesticide combinations containing imidacloprid were safe to *O. bicornis*. The incorporation of tests specifically intended to detect sublethal effects in bee risk assessment schemes should be an urgent priority. In this way, the effects of pesticide exposure on the dynamics of bee populations in agroecosystems will be better assessed.

## Introduction

Bees, both wild and managed, play an essential role in crop pollination and food production stability^1–3^. Yet, especially in intensively farmed areas, bee populations often face adverse environmental conditions, including destruction of nesting habitats, scarcity of floral resources and intensive pesticide presence^4–7^. Before being approved for commercial use, pesticides undergo a risk assessment process to ensure they do not pose unacceptable threats to non-target organisms, including bees. However, current risk assessment schemes in the US and Europe, have an important limitation: they test for the effects of single pesticides^8,9^, even though bees in agricultural areas are likely to be exposed to combinations of pesticides^10,11^. Multiple residues have been found in the pollen and nectar of flowering crops^12–14^, wild flowers growing in agricultural field margins^15–17^, food provisions of honey bees^18,19^ and wild bees^20–22^, and on the body of honey bees^18,23^ and bumblebees^10,24^. Focusing on single compounds may underestimate the risks of pesticide use on bees because the exposure to multiple compounds may result not only in additive but also in synergistic adverse effects^22,25–29^.

Adult bees may be exposed to pesticides through various routes (inhalation, contact, oral). To simulate oral exposure, most studies expose bees to contaminated “nectar” (sugar-water solution laced with the desired amounts of pesticide). However, adult bees also ingest considerable amounts of pollen^30,31^. Because pollen from flowers growing in agricultural areas has been shown to contain pesticide residues^12,13,15^, exposure via pollen should be tested in combination with exposure via nectar.

Traditionally, most bee ecotoxicological studies assess lethal and/or sublethal effects following short-term (acute) exposure^32,33^. However, due to pesticide persistence in the environment, bees in field conditions are often exposed for long periods of time (chronic exposure)^15^. Exposure to very low doses for long periods of time may result in lethal effects due to cumulative toxicity^34^.

In this study, we chronically exposed females of a solitary bee to combinations of two insecticides and a fungicide via syrup and pollen ingestion. In an attempt to mimic field-realistic conditions, we used pesticide concentrations found in pollen and nectar of melon flowers in commercial open-field plantations in central Spain (Table 1). Melons are widely cultivated worldwide (1,245,841 ha in 2016^35^). They are frequently sprayed with insecticides to control mainly aphids and whiteflies and with fungicides during bloom to control powdery mildew and other fungal diseases^36,37^. Melons require bee pollination^38,39^ and farmers often rent *Apis mellifera* L. hives to increase pollination levels. Melon flowers are also visited by a wide variety of wild bee species^5,39–41^.

**Table 1:** Concentration and occurrence of Acetamiprid, Imidacloprid and Myclobutanil in the pollen and nectar of melon flowers from 5 commercial melon fields near Madrid (Spain).

Pesticide	Class	N of fields sprayed	Mean (±SE) concentration (ppb) in melon flowers		Occurrence (% samples)	Days between field application and pollen/nectar surveys	Analytical technique
			pollen	nectar			
Acetamiprid (A)	Neonicotinoid insecticide	5	482.93 ± 215.85	6.41 ± 1.45	100.0%	2–11	HPLC-QQQ
Imidacloprid (I)	Neonicotinoid insecticide	4	369.36 ± 186.31	15.34 ± 7.62	66.7%	45–71^a^	HPLC-QQQ
Myclobutanil (M)	Triazole fungicide	4	0	5.58 ± 0.70	26.7%	2–15	GC-QQQ

(See details of analytical techniques in Supplementary Information).

^a^Timing of application unknown for one of the 5 fields.

HPLC-QQQ: high performance liquid chromatography with triple-quadrupole mass spectrometer detector; GC-QQQ: gas chromatography with triple-quadrupole mass spectrometer detector.

Our aim was to establish whether the exposure to combinations of a cyano-substituted neonicotinoid (acetamiprid), a nitro-substituted neonicotinoid (imidacloprid) and a triazole fungicide (myclobutanil) separately and in binary and ternary mixtures causes lethal and/or sublethal effects in the solitary bee *Osmia bicornis* L. (Megachilidae). We assessed longevity, syrup and pollen consumption, ovary maturation and thermogenesis. Based on previous studies on neonicotinoid-fungicide combinations^26,42,43^, we expected a greater synergistic effect for acetamiprid than imidacloprid.

## Results

### Survival and longevity

**Figure 1:**
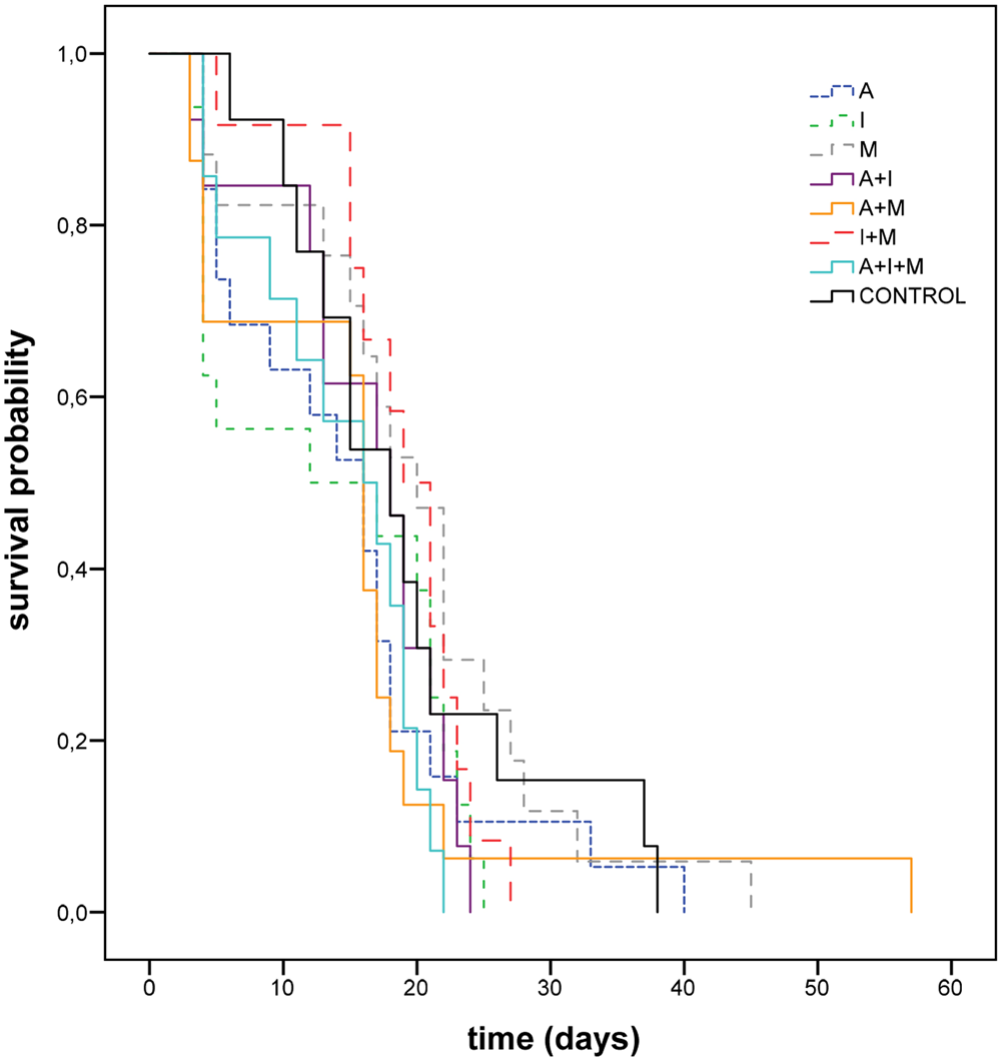
Cumulative survival probability in *O. bicornis* females chronically exposed to eight pesticide oral treatments at field-realistic concentrations. A: acetamiprid, I: imidacloprid, M: myclobutanil (Log Rank test: *p* < 0.05).

Chronic exposure to the three pesticides and their mixtures at field-realistic concentrations had no effect on survival and longevity of *Osmia* females. Cumulative survival curves did not differ significantly among treatments (Log Rank test: F = 6.53, df = 7, *p = *0.42, Fig. 1). Longevity (overall mean = 16.32 ± 0.86 days) did not significantly differ among treatments (GLM: F = 1.22, df = 7, *p* = 0.30), and was not influenced by body size (GLM: F = 0.03, df = 1, *p* = 0.89). There were no differences among treatments in body size (ANOVA: F = 0.746; df = 7; *p* = 0.63, Table 2).

**Table 2:** Body weight and amount of active ingredient ingested via syrup and pollen in *O. bicornis* females exposed to various pesticide combinations (treatments) throughout their adult life span (chronic exposure).

Treatment	n bees	Body weight (mean ± SE mg)	Acetamiprid (mean ± SE ng bee^−1^)				Imidacloprid (mean ± SE ng bee^−1^)				Myclobutanil (mean ± SE ng bee^−1^)
			Syrup	Pollen		Total	Syrup	Pollen		Total	Syrup
				Period 1	Period 2			Period 1	Period 2		
A	20	70.67 ± 1.87	2.88	7.41	0.34	10.63					
I	16	68.93 ± 1.58					1.63	8.16	0.55	8.71	
M	17	71.09 ± 2.03									3.42
A + I	13	69.08 ± 2.85	0.58	4.70	0.04	5.33	1.40	3.60	0.03	3.63	
A + M	16	72.31 ± 1.72	3.34	14.47	0.27	18.08					2.91
I + M	12	71.50 ± 2.37					1.59	8.29	0.28	8.57	0.58
A + I + M	14	66.72 ± 2.16	0.53	6.72	0.17	7.41	1.27	5.14	0.13	5.27	0.46
CONTROL	13	68.19 ± 3.01									

A: acetamiprid, I: imidacloprid, M: myclobutanil. Period 1: first week; Period 2: remainder of the bioassay.

### Syrup and pollen consumption

**Figure 2:**
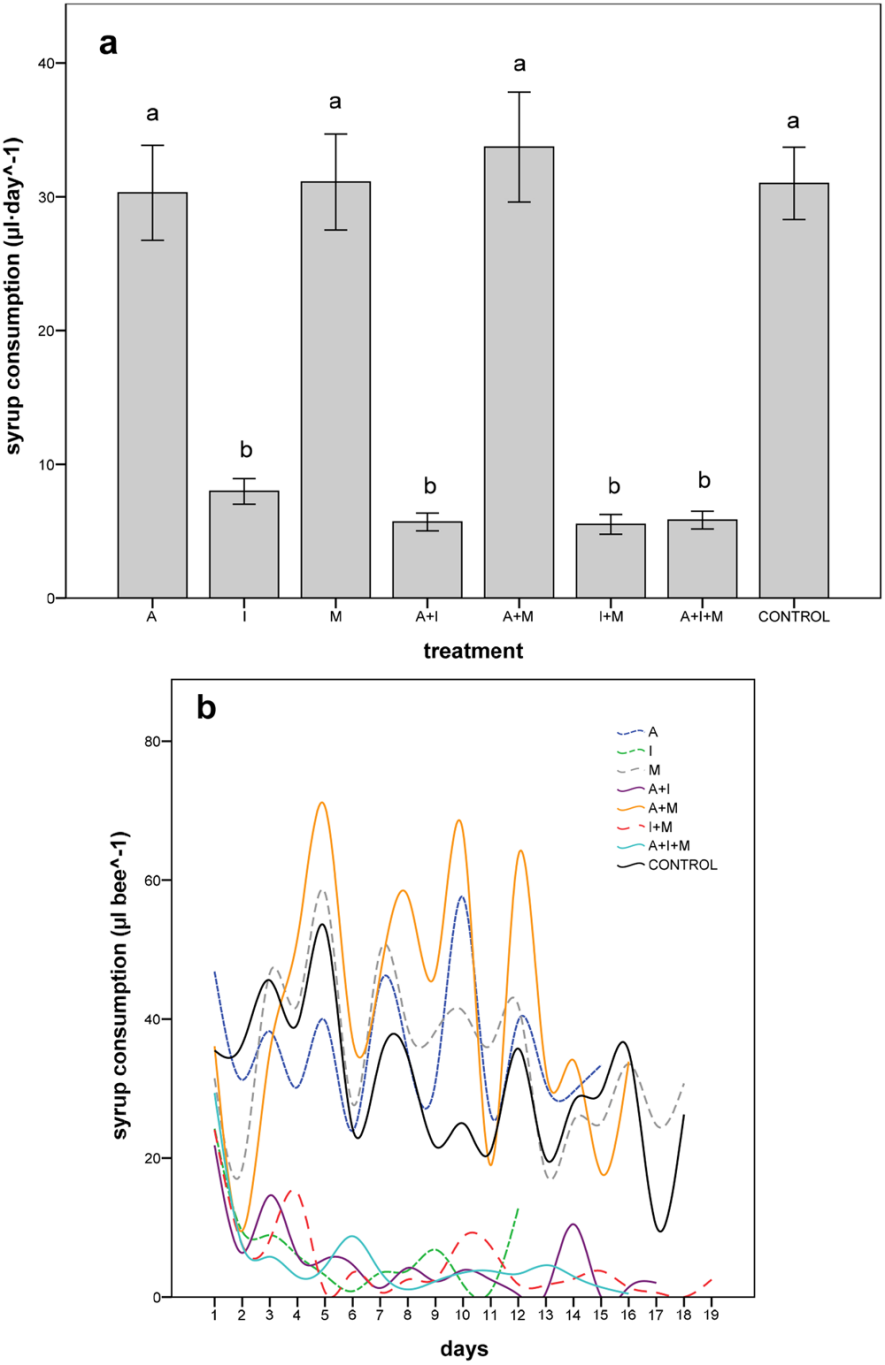
Mean (±SE) daily syrup consumption (µl day^−1^) (**a**) and syrup consumption (µl bee^−1^) over time until 50% mortality (**b**) in *O. bicornis* females chronically exposed to eight pesticide oral treatments at field-realistic concentrations. A: acetamiprid, I: imidacloprid, M: myclobutanil. Means with the same letter are not significantly different (Fisher´s LSD post hoc; *p* < 0.05).

In all treatments containing imidacloprid (I, A + I, I + M, A + I + M), bees consumed approximately 80% less syrup per day than in the rest of the treatments including the control (GLM: F = 38.16, df = 7, *p* < 0.001; Fig. 2a). The effect of imidacloprid on syrup consumption began on day 2 (Fig. 2b); differences among treatments were not significant on day 1; (GLM: F = 0.52, df = 7, *p* = 0.82). Body size affected syrup consumption (GLM: F = 4.22, df = 1, *p* = 0.04), with larger bees tending to consume more syrup in all the treatments except M and A + I.

**Figure 3:**
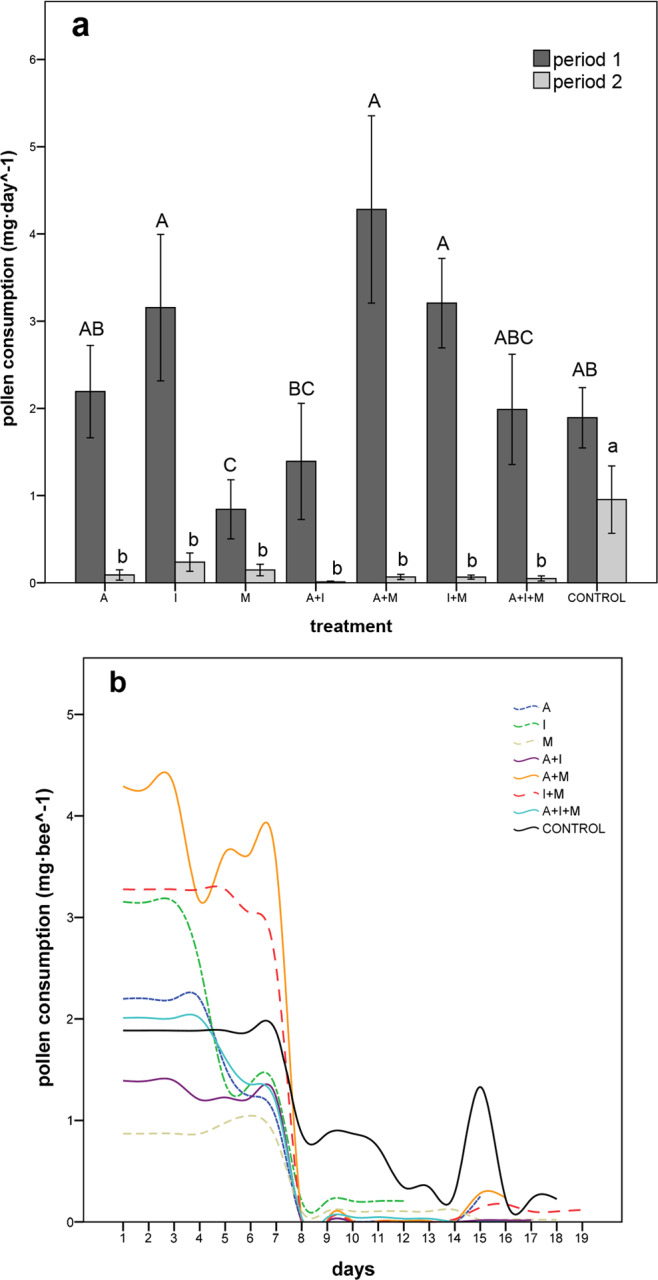
Mean (±SE) daily pollen consumption (mg day^−1^) (**a**) and pollen consumption (mg bee^−1^) over time until 50% mortality (**b**) in *O. bicornis* females chronically exposed to eight pesticide oral treatments at field-realistic concentrations. A: acetamiprid, I: imidacloprid, M: myclobutanil. Period 1: first week; Period 2: remainder of the bioassay. Means with the same letter are not significantly different (Fisher´s LSD post hoc; *p* < 0.05).

Daily pollen consumption ranged between 1 and 4 mg per bee during the first week of exposure, and then abruptly decreased in all treatments (Fig. 3b). We found significant differences between these two periods (GLMM: F = 137.97, df = 1, *p* < 0.001) and among treatments (GLMM: F = 3.62, df = 7, *p* = 0.002), as well as a significant interaction between period and treatment (GLMM: F = 3.41, df = 7, *p* = 0.002) (Fig. 3a). During period 1, only bees of treatment M consumed significantly less pollen than control bees whereas, in period 2, pollen consumption was significantly low in all treatments compared to the control (Fig. 3a). Body size had no effect on pollen consumption (GLMM: F = 0.30, df = 1, *p* = 0.59).

The total amounts of pesticide ingested via syrup and pollen by bees of each treatment throughout the entire exposure are reported in Table 2.

### Thoracic temperature

**Figure 4:**
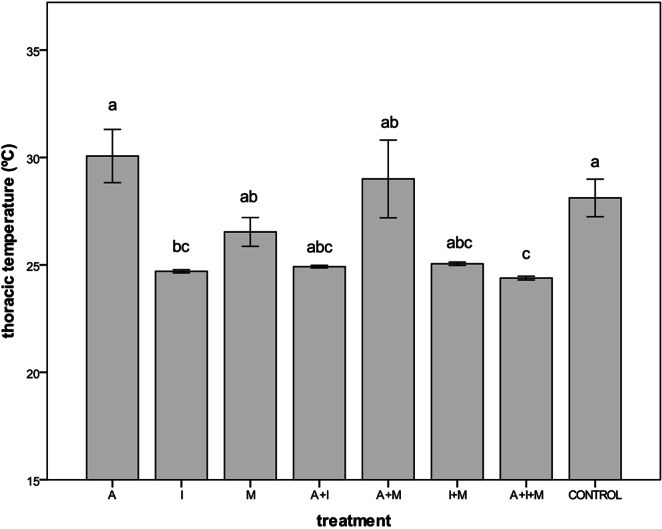
Mean (±SE) thoracic temperature (°C) in *O.bicornis* females after 17 days of chronic exposure to eight pesticide oral treatments at field-realistic concentrations. A: acetamiprid, I: imidacloprid, M: myclobutanil. Means with the same letter are not significantly different (Kruskal-Wallis test followed by Dunn’s post hoc; *p* < 0.05).

Thoracic temperature significantly differed among treatments (Kruskal-Wallis: χ^2^ = 38.83, df = 7, *p* < 0.001, Fig. 4). The lowest temperatures were registered in bees of the four treatments containing imidacloprid (I) although only treatments I and A + I + M differed significantly from the control (Fig. 4). Low temperatures were accompanied by clear signs of apathy in bees of these four treatments (see Supplementary Video S1). These signs were not observed in any of the other treatments.

### Ovary maturation

No significant differences were found in mean basal oocyte length among treatments (GLM: F = 1.45, df = 7, *p* = 0.20). Oocyte length was positively related to body size in all treatments (GLM: F = 24.7, df = 1, *p* < 0.00).

## Discussion

Bees in agroecosystems are chronically exposed to combinations of pesticides^10,11^. However, the effects of this exposure scenario are not well understood because most laboratory studies test acute exposure to single products at concentrations that often are not field-realistic^44^. In addition, most studies addressing oral exposure only consider the nectar route, overlooking pesticide ingestion via pollen consumption. We tested chronic exposure to pollen and syrup contaminated with field-measured concentrations of pesticide combinations found in pollen and/or nectar in commercial melon plots. To our knowledge, this is the first time pesticide exposure via pollen is tested in adult solitary bees. Two previous studies have exposed *Osmia* larvae to neonicotinoids via pollen^45,46^. At field-realistic doses, these studies did not find any effects on larval survival or adult performance.

With the exception of myclobutanil, which was not detected in pollen, pesticide concentrations (ppb) were one or two orders of magnitude higher in pollen than in nectar of melon flowers. Other studies measuring pesticide levels from pollen and nectar have found similar results^13–15,18^. However, because solitary bee adults consume much greater amounts of nectar than pollen (ca. 93% of total food weight consumed by bees in our study was via syrup), the amounts of active ingredient ingested per bee in our study were similar via pollen and via syrup. This is important because some laboratory studies expose bees via syrup to pesticide concentrations found in pollen^29,47^, thus exposing bees to doses presumably higher than those encountered by bees under field conditions.

Contrary to other studies testing mixtures of neonicotinoid insecticides and triazole fungicides on *O. bicornis*^22,27^ and other bee species^26,27,42,43^ we did not find synergistic effects between these two classes of pesticides. This discrepancy may be due to the identity of the compounds involved. In general, cyano-substituted neonicotinoids (including acetamiprid and thiacloprid) show higher synergism than nitro-substituted neonicotinoids (including imidacloprid, clothianidin and thiamethoxam)^26,43^. However, even within these two subgroups of neonicotinoids differences among compounds have been found. In agreement with our results, Thompson *et al*.^42^ did not observe synergism between triazole fungicides and imidacloprid but they found synergism between these fungicides and two other nitro-substituted neonicotinoids (clothianidin and thiamethoxam) in honeybees. Differences between our results and those of other studies can also be explained by differences in the route of exposure. Iwasa *et al*.^26^ and Biddinger *et al*.^43^ found synergism between triazole fungicides and acetamiprid applied topically, as opposed to orally in our study. Finally, differences between our results and those of other studies may also be explained by differences in the concentrations to which bees were exposed. Synergism between triazole fungicides and neonicotinoids has been shown to be concentration-dependent^42^. In our study, the dose of myclobutanil consumed by *O. bicornis* throughout their lifespan in treatment A + M was 2.91 ng bee^−1^. This dose is 8–153 times lower than the triazole fungicide doses tested in Thompson *et al*.^42^ (propiconazole: 22.4 ng bee^−1^; tebuconazole: 447 ng bee^−1^). In treatments containing imidacloprid (I + M and A + I + M), due to the inhibitory effect of this compound on syrup feeding, the levels of myclobutanil ingested by bees were even lower. Overall, the doses of myclobutanil ingested by *O. bicornis* in our study are ca. 1000 times lower than the lethal doses estimated by Han *et al*.^48^ in *Apis cerana* F. (acute oral toxicity: LD_50_ = 2,154 ng bee^−1^ and LD_5_ = 1,085 ng bee^−1^).

Following emergence, *Osmia* females undergo a short period (2–5 days) prior to initiating nesting activities^49,50^. During this period, females consume pollen^30^ and complete ovary maturation^49,51,52^. The high levels of pollen consumption recorded during the first seven days of exposure in our study are congruent with the results of Cane^30^. During this phase (period 1), treatment M showed significantly lower pollen consumption than control bees. On first sight, the M result may seem surprising because pollen in this treatment was not contaminated (no myclobutanil residues were found in the pollen of melon flowers) (Table 1). However, this treatment resulted in the highest ingestion of myclobutanil via syrup (Table 2). We also found differences in pollen consumption during the second week following exposure. In this case, all treatments yielded significantly lower feeding levels than the control. Nevertheless, the differences found in pollen consumption among treatments did not result in differences in ovary maturation, which did not vary across treatments. This in contrast to a previous study that found a lower ovary maturation in *Osmia* females co-exposed to clothianidin and propiconazole^22^. Again, this discrepancy may be explained by the different compounds, as well as by the concentrations tested. Because they were interested in exposure right after fungicide application to a flowering crop, Sgolastra *et al*.^53^ tested propiconazole at the field application rate (62.5 mg L^−1^). By contrast, we tested myclobutanil at the concentration found in the nectar of melon flowers 2–15 days after application (5.58 µg L^−1^). Under field conditions, pesticides degrade over time and this process has not been considered in our laboratory study. At any rate, toxic effects are expected to be greater right after application and therefore the concentrations used in our study do not represent the worst case scenario for bees. Studies evaluating pesticide degradation under field conditions are needed to better understand the extent of chronic exposure of bees to pesticides in agricultural landscapes.

Imidacloprid had a clear inhibitory effect on syrup consumption. On the other hand, we did not detect any changes in pollen consumption, possibly due to overall low amounts of pollen ingested in all treatments. *Osmia bicornis* females ingested approximately 80% less syrup in all treatments containing imidacloprid compared to the other treatments, including the control. As a result, the dose of imidacloprid (alone and in mixtures) ingested by *O. bicornis* females throughout their life-span was ca. 4–9 ng. This amount is 1.4–6.8 times lower than the acute oral LD_50_ reported in honey bees (13 ng bee^−1^ ^54^) and bumblebees (27 ng bee^−1^ ^54^). For the same reason, the amounts of acetamiprid and/or myclobutanil ingested by bees in A + I, I + M and A + I + M were also reduced by 80% when compared to treatments containing acetamiprid and myclobutanil but not imidacloprid (Table 2). Feeding suppression following exposure to this neonicotinoid has also been reported in *A. mellifera*^29^ and *Bombus terrestris* L.^47,55,56^. Because bees cannot taste neonicotinoids^57^, feeding suppression is likely to be due to the toxicity of the neonicotinoid rather than repellence. Kessler *et al*.^57^ found that honey bees and bumblebees preferred syrup containing imidacloprid to control solutions, even though ingestion of this compound caused them to eat less syrup overall. We found feeding suppression in *O. bicornis* exposed to imidacloprid at doses as low as 0.2–0.5 ng bee^−1^ day^−1^. In agreement with our results, the anti-feeding response caused by imidacloprid ingestion has been shown to be greater under chronic exposure^55,56^.

Feeding suppression in imidacloprid-exposed *O. bicornis* was accompanied by decreased thoracic temperature and apathy. These symptoms could be caused by a general lack of energy due to low feeding levels. However, there is accumulating evidence that imidacloprid directly affects muscular activity. A transcriptome study showed significant down-regulation of twenty-two genes related to muscle function in imidacloprid (10 ppb) treated bees^58^. Thoracic muscles (the largest in a bee body) are involved in thermoregulation and flight. Other studies document disrupted thermogenic capacity in honey bees^59^ and bumblebees^60^ following exposure to imidacloprid and thiamethoxam. These studies show that ingestion of small doses of neonicotinoids results in an initial short-term stimulation followed by decreased thoracic temperature the day after exposure^59^. Other studies have shown that acute exposure to field-realistic doses of neonicotinoids causes excitation (hyperactivity), whereas chronic exposure causes depression (hypoactivity) and impairs flight ability^61–64^. In agreement with our results, Crall *et al*.^65^ show that workers orally exposed to 6 ppb of imidacloprid were less active compared to control workers. Studies in bumblebees at the colony level have demonstrated that exposure to imidacloprid impairs colony thermoregulation and alters nursing behaviour and social and spatial dynamics^65^ and decrease pollen intake^25,66^.

Our results show clear differences between the two neonicotinoids tested. Acetamiprid yielded no negative effects, even though the amounts of this compound ingested in treatments A and A + M were twice as high as amounts of imidacloprid ingested in any of the treatments containing imidacloprid. Other studies have found acetamiprid to be less toxic to bees than imidacloprid^26^. These findings are particularly relevant in the context of the Integrated Pest and Pollinator Management (IPPM)^67^, which aims to include pollinator health into the Integrated Pest Management (IPM) paradigm. Whenever effective non-chemical alternatives are not available, IPPM advocates for the use of pesticides that are less toxic to bees and other beneficial insects. IPPM relies on information on lethal and sublethal toxicity of commonly applied pesticides to wild and managed bees.

Our results also have important consequences for bee risk assessment. Current bee risk assessment schemes rely on estimates of LD_50_ (dose at which half of the population dies) at 48 h following exposure. None of the compounds or mixtures tested in our study resulted in increased mortality. Therefore, if we had considered only lethal effects, we would have wrongly concluded that, at field-realistic doses, all compounds and mixtures tested were safe to bees. Yet, some of our treatments profoundly impaired thermoregulation and bee activity. It is important to note that this effect was not restricted to the immediate post-exposure period, since thoracic temperature was measured on the 17^th^ day of exposure. Although, the ecological consequences of this effect should be confirmed in field conditions, we conclude that incorporating tests specifically intended to detect sublethal effects into risk assessment schemes is essential to evaluate the impact of pesticide exposure on the dynamics of bee populations in agroecosystems.

## Methods

### Bee population and test conditions

All tests were conducted with newly-emerged females of *O. bicornis* L., a cavity-nesting solitary bee. This species has not been recorded visiting melon flowers but we decided to work with this species for various reasons. First, it can be easily reared under controlled conditions; second, it has been proposed by the European Food Safety Authority as a test species for risk assessment^68^; third, a fair amount of information is available on *Osmia* ecotoxicology^22,27,43,46,69–73^ and the available evidence indicates a higher vulnerability to pesticides than in honey bees and bumblebees^20,21,27^.

**Figure 5:**
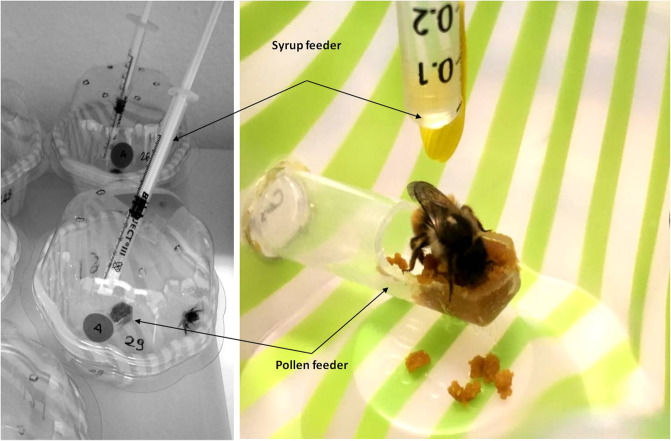
Individual cages and close-up of syrup and pollen feeders.

*Osmia bicornis* cocoons from a population reared in a pesticide-free area of the Kazimierz Landscape Park (Poland) were shipped to the laboratory of Agricultural Entomology at the University of Bologna (Italy) in January 2018 and kept at 3–4 °C until May 2018. At that time, large cocoons (presumed to contain females) were incubated at 22–23 °C until emergence. A previous study showed that emergence time influences sensitivity to pesticides in *O. bicornis* (females taking longer to emerge are more sensitive^53^). For this reason, we only used bees that emerged over two consecutive days during the peak of the emergence period (days 4–5). Upon emergence (<24 h) bees were transferred to a Plexiglas holding cage (50 × 50 × 50 cm) for ca. 4 hours to allow them to deposit the meconium. Then, bees were individually caged in plastic ice cream cups (diameter: 5.5–8 cm; height: 7 cm) with transparent lids perforated with a pin to allow air exchange. Each cup contained a syrup feeder and a pollen feeder. The syrup feeder was a 1-ml calibrated syringe (Tuberculin Beroject® III, Beromed; accuracy: 0.01 ml) inserted through the lid. A petal of *Euryops* (Asteraceae) was attached to the tip of the syringe to enhance location of the feeder by the bee (Fig. 5). The pollen feeder was a 1.5-ml Eppendorf tube inserted through the side of the cage with the upper half of the bottom cut off (Fig. 5). Bees were maintained at 23.6 ± 0.3 °C and 50–60% relative humidity. Cups were kept under natural light conditions throughout the experiment but direct sunlight exposure was avoided to reduce pesticide degradation^74^.

### Treatments

Previous analysis of the pollen and nectar of melon flowers from 5 commercial fields southeast of Madrid, Spain, yielded 19 pesticides (acetamiprid, imidacloprid, oxamyl, metalaxil-m, chlorpyrifos, abamectin, azoxystrobin, myclobutanil, boscalid, flonicamid, atrazina, quinomethionato, clorantraniliprol, difenoconazole, kresoxim-methyl, chlorothalonil, thiacloprid, *alfa-*cypermethrin, quinoxyfen). Because it was not feasible to test so many compounds, we decided to work with three of them: the triazole fungicide, myclobutanil, and two neonicotinoid insecticides, imidacloprid and acetamiprid (Table 1). These three compounds were selected because: (1) they are the pesticides most commonly applied to melon fields in the study area; (2) their occurrence in the pollen/nectar samples was very high; (3) the two neonicotinoids have different detoxification pathways and differ in their toxicity to bees^75^; and (4) several studies have found synergistic effects between mixtures of neonicotinoid insecticides and triazole fungicides^22,26,27,42,43^.

We exposed bees to the mean active ingredient concentrations found in the nectar and pollen of melon flowers in commercial fields (Table 1). Females emerging on any given day were evenly distributed among eight treatments: control (CON), acetamiprid (A), imidacloprid (I), myclobutanil (M) and the mixtures A + I, A + M, I + M, A + I + M. Each treatment group received the specific food for the entire test period, i.e. until the natural death of the bees.

The syrup was prepared by diluting sucrose in water (33% w/w). Honey bee pollen pellets were obtained from an organic beekeeper (Bona Mel^®^) and stored at 3–4 °C until use. Pellets were then ground with a coffee grinder and mixed with distilled water (pollen/water 3:1 w/w) to obtain a single uniform pollen source. Although honeybee- collected pollen could be a potential source of pathogens^76,77^, we did not irradiate the pollen pellets^78^. Nonetheless, we are confident that this did not affect our results because mean longevity of control bees in our study (19 days) was similar to mean longevity recorded in previous *O. bicornis* laboratory studies (17 days) in which bees were only fed syrup^22^. This longevity is also similar to mean life span of adult *Osmia* females nesting in field and semi-flied conditions (17.5–24 days)^49,50,79^.

Stock solutions of each pesticide were prepared by diluting 500 mg of Epik^®^ (acetamiprid, 20% w/w), 100 µl of Confidor^®^ (imidacloprid, 20% w/v) and 100 µl of Systhane Forte^®^ (myclobutanil, 24% w/v) in 50 ml of purified distilled water. These solutions were diluted in the syrup or in the distilled water used for the pollen preparation to reach the desired concentrations identified in the pollen and/or nectar of the melon flowers (Table 1).

### Syrup consumption, pollen consumption and longevity

Cups were inspected daily to monitor syrup consumption (assessed by checking the level of syrup in the calibrated syringe) and bee mortality. Pollen consumption was assessed once a week and whenever a bee died. For each cup, we weighed the pollen remaining in the Eppendorf tube along with any pollen crumbs scattered over the bottom of the holding cage with an analytical scale (accuracy = 0.0001 g). Average daily pollen consumption was estimated by dividing pollen consumption by the number of days elapsed between measurements. Eight additional containers with syrup and pollen feeders but without bees were used as controls to measure and account for potential evaporation from the syrup and pollen sources. Five additional five cages without bees were used to measure the evaporation of the pollen crumbs scattered over the bottom of the holding cage. Syrup was renewed every 3–4 days and pollen once a week.

Bees that had not begun feeding by the fourth day of exposure were discarded. Sample sizes in each treatment are shown in Table 2. At the end of the experiment, we measured the head width of each bee under a stereomicroscope at 240x as a proxy of body size^80^.

### Thoracic temperature

Some bees showed clear signs of apathy (see Supplementary Video recordings S1). For this reason, we decided to measure thoracic temperature as a proxy of muscular activity. Thermogenesis in bees is mainly achieved by shivering of the flight muscles^81^. We used a compact thermal imaging camera FLIR e60bx (320 × 240 pixels; range: −20 °C to 120 °C; sensitivity: <0.045 °C at 30 °C) to take thermal photographs of the bees in their cages in a dark room at 24.6 °C. These measures were taken on the 17^th^ day of exposure in 6 bees per treatment.

### Ovary maturation

Upon emergence from the cocoon, *Osmia* females take about 3 days to fully mature their ovaries^49,51,52^. On day 3 of the exposure phase, we took 14 bees per treatment and froze them at −24 °C. These bees were later dissected in Ringer’s physiological solution (NaCl 9 g, KCl 0.2 g, NaHCO_3_ 0.2 g, CaCl_2_ 0.2 g in 1 litre of distilled water), and the length of the most mature oocyte in each of the 6 ovarioles was measured under a stereo microscope at 500x (precision, ±0.01 mm). We use the mean length of these 6 oocytes as a measure of ovary maturation. At the end of the experiment, the head width of each bee was measured as described above.

### Statistical analysis

We used Gehan-Breslow Kaplan-Meier (K-M) survival analysis with pairwise multi comparison procedures (Log-Rank Test, *p* < 0.05) to compare survival curves among treatments. We used general linear models (GLM) to analyze the effect of treatment on longevity (square-root transformed), mean daily syrup consumption and ovary maturation (log-transformed). Body size was included as covariate in all these models and pairwise comparisons were conducted with the Fisher´s LSD test (*p* < 0.05). Pollen consumption followed a clear two-phase temporal pattern (see results). For this reason, to analyse the effect of treatment on mean daily pollen consumption (log-transformed), we used a general linear mixed model (GLMM) with treatment (fixed factor), period (fixed factor repeated within subjects), their interaction, and body size as a covariate. Means were separated using Fisher’s LSD test (*p* < 0.05). Thoracic temperature data were not normally distributed and could not be appropriately transformed. For this reason, we used non-parametric Kruskal-Wallis followed by Dunn’s multiple pairwise comparisons (*p* < 0.05) to establish differences in thoracic temperature among treatments.

## Supplementary information

Suplementary Information

Video S1

## Data Availability

Data is available upon request to the main author.

## References

[1] Klein AM (2007). Importance of pollinators in changing landscapes for world crops. Proc. R. Soc. B Biol. Sci..

[2] Fontaine C, Dajoz I, Meriguet J, Loreau M (2006). Functional diversity of plant–pollinator interaction webs enhances the persistence of plant communities. PLoS Biol..

[3] Garibaldi LA, Aizen MA, Klein AM, Cunningham SA, Harder LD (2011). Global growth and stability of agricultural yield decrease with pollinator dependence. Proc. Natl. Acad. Sci..

[4] Potts SG (2010). Global pollinator declines: trends, impacts and drivers. Trends Ecol. Evol..

[5] Kremen C, Williams NM, Thorp RW (2002). Crop pollination from native bees at risk from agricultural intensification. Proc. Natl. Acad. Sci. USA.

[6] Sánchez-Bayo F, Wyckhuys KAG (2019). Worldwide decline of the entomofauna: A review of its drivers. Biol. Conserv..

[7] Goulson D, Nicholls E, Botías C, Rotheray EL (2015). Bee declines driven by combined stress from parasites, pesticides, and lack of flowers. Science (80-.)..

[8] EPPO (European and Mediterranean Plant Protection Organization). *Enviromental risks assesment scheme for plant protection products. Chapter 10: honeybees*. *EPPO Bulletin* **40** (2010).

[9] U.S. Environmental Protection Agency (USEPA) Health Canada Pest Management Regulatory Agency (PMRA) and California Departament of Pesticide Regulatio. *Guidance for Assessing Pesticide Risks to Bees*. *Office of Pesticide Programs USEPA, Health Canada PMRA, CDPR* (2014).

[10] Botías C, David A, Hill EM, Goulson D (2017). Quantifying exposure of wild bumblebees to mixtures of agrochemicals in agricultural and urban landscapes. Environ. Pollut..

[11] Tosi S, Costa C, Vesco U, Quaglia G, Guido G (2018). A 3-year survey of Italian honey bee-collected pollen reveals widespread contamination by agricultural pesticides. Sci. Total Environ..

[12] Bonmatin JMB, Archand PAM, Harvet RC, Oineau IM (2005). Quantification of imidacloprid uptake in maize crops. J. Agric. Food Chem..

[13] Dively GP, Kamel A (2012). Insecticide residues in pollen and nectar of a cucurbit crop and their potential exposure to pollinators. J. Agric. Food Chem..

[14] Stoner KA, Eitzer BD (2012). Movement of soil-applied imidacloprid and thiamethoxam into nectar and pollen of squash (Cucurbita pepo). PLoS One.

[15] Botías C (2015). Neonicotinoid residues in wildflowers, a potential route of chronic exposure for bees. Environ. Sci. Technol..

[16] David A (2016). Widespread contamination of wildflower and bee-collected pollen with complex mixtures of neonicotinoids and fungicides commonly applied to crops. Environ. Int..

[17] Tsvetkov N (2017). Chronic exposure to neonicotinoids reduces honey bee health near corn crops. Science.

[18] Mullin CA (2010). High levels of miticides and agrochemicals in north American apiaries: implications for honey bee health. PLoS One.

[19] Porrini C (2016). The status of honey bee health in Italy: results from the nationwide bee monitoring network. PLoS One.

[20] Rundlöf M (2015). Seed coating with a neonicotinoid insecticide negatively affects wild bees. Nature.

[21] Woodcock BA (2017). Country-specific effects of neonicotinoid pesticides on honeybees and wild bees Authors. Science (80-.)..

[22] Sgolastra F (2018). Combined exposure to sublethal concentrations of an insecticide and a fungicide affect feeding, ovary development and longevity in a solitary bee. Proc. R. Soc. B Biol. Sci..

[23] Kiljanek T (2017). Multiple pesticide residues in live and poisoned honeybees – Preliminary exposure assessment. Chemosphere.

[24] David A, Botías C, Abdul-Sada A, Goulson D, Hill EM (2015). Sensitive determination of mixtures of neonicotinoid and fungicide residues in pollen and single bumblebees using a scaled down QuEChERS method for exposure assessment. Anal. Bioanal. Chem..

[25] Gill RJ, Ramos-Rodriguez O, Raine NE (2012). Combined pesticide exposure severely affects individual- and colony-level traits in bees. Nature.

[26] Iwasa T, Motoyama N, Ambrose JT, Roe RM (2004). Mechanism for the differential toxicity of neonicotinoid insecticides in the honey bee, Apis mellifera. Crop Prot..

[27] Sgolastra F (2017). Synergistic mortality between a neonicotinoid insecticide and an ergosterol-biosynthesis-inhibiting fungicide in three bee species. Pest Manag. Sci..

[28] Sgolastra F (2018). Lethal effects of Cr(III) alone and in combination with propiconazole and clothianidin in honey bees. Chemosphere.

[29] Zhu YC, Yao J, Adamczyk J, Luttrell R (2017). Feeding toxicity and impact of imidacloprid formulation and mixtures with six representative pesticides at residue concentrations on honey bee physiology (Apis mellifera). PLoS One.

[30] Cane JH (2016). Adult pollen diet essential for egg maturation by a solitary Osmia bee. J. Insect Physiol..

[31] Cane JH, Dobson HEM, Boyer B (2017). Timing and size of daily pollen meals eaten by adult females of a solitary bee (Nomia melanderi) (Apiformes: Halictidae). Apidologie.

[32] Decourtye A (2005). Comparative sublethal toxicity of nine pesticides on olfactory learning performances of the honeybee Apis mellifera. Arch. Environ. Contam. Toxicol..

[33] Laurino D, Porporato M, Patetta A, Manino A (2011). Toxicity of neonicotinoid insecticides to honey bees: laboratory tests. Bull. Insectology.

[34] Rondeau G (2014). Delayed and time-cumulative toxicity of imidacloprid in bees, ants and termites. Sci. Rep..

[35] FAO. Food and Agriculture Organization of the United Nations. FAOSTAT. at, http://www.fao.org/faostat/en/#data/QC (2018).

[36] Duncan, J. & Ewing, J. Specialty melon production for small and direct-market growers. *ATTRA Sustain. Agric*. 1–16 (2015).

[37] Khetereli, A., Baramidze, V. & Kushad, M. Chemical management guidelines for control of pests and diseases of vegetables and melons in Georgia. Strengthening Extension and Advisory Services (2016).

[38] Bomfim Isac, Freitas Breno, de Aragão Fernando, Walters Stuart (2016). Pollination in Cucurbit Crops. Handbook of Cucurbits.

[39] Tschoeke PH, Oliveira EE, Dalcin MS, Silveira-Tschoeke MCAC, Santos GR (2015). Diversity and flower-visiting rates of bee species as potential pollinators of melon (Cucumis melo L.) in the Brazilian Cerrado. Sci. Hortic. (Amsterdam)..

[40] Winfree R, Williams NM, Gaines H, Ascher JS, Kremen C (2008). Wild bee pollinators provide the majority of crop visitation across land-use gradients in New Jersey and Pennsylvania, USA. J. Appl. Ecol..

[41] Rodrigo Gómez S, Ornosa C, Selfa J, Guara M, Polidori C (2016). Small sweat bees (Hymenoptera: Halictidae) as potential major pollinators of melon (Cucumis melo) in the Mediterranean. Entomol. Sci..

[42] Thompson HM, Fryday SL, Harkin S, Milner S (2014). Potential impacts of synergism in honeybees (Apis mellifera) of exposure to neonicotinoids and sprayed fungicides in crops. Apidologie.

[43] Biddinger DJ (2013). Comparative toxicities and synergism of apple orchard pesticides to Apis mellifera (L.) and Osmia cornifrons (Radoszkowski). PLoS One.

[44] Carreck NL, Ratnieks FLW (2014). The dose makes the poison: have “field realistic” rates of exposure of bees to neonicotinoid insecticides been overestimated in laboratory studies?. J. Apic. Res..

[45] Nicholls E, Fowler R, Niven JE, Gilbert JD, Goulson D (2017). Larval exposure to field-realistic concentrations of clothianidin has no effect on development rate, over-winter survival or adult metabolic rate in a solitary bee, Osmia bicornis. PeerJ.

[46] Abbott VA, Nadeau JL, Higo HA, Winston ML (2008). Lethal and sublethal effects of imidacloprid on Osmia lignaria and clothianidin on Megachile rotundata (Hymenoptera: Megachilidae). J. Econ. Entomol..

[47] Laycock I, Lenthall KM, Barratt AT, Cresswell JE (2012). Effects of imidacloprid, a neonicotinoid pesticide, on reproduction in worker bumble bees (Bombus terrestris). Ecotoxicology.

[48] Han W (2018). Acute toxicity and sublethal effects of myclobutanil on respiration, flight and detoxification enzymes in Apis cerana cerana. Pestic. Biochem. Physiol..

[49] Sgolastra F (2016). Pre-wintering conditions and post-winter performance in a solitary bee: does diapause impose an energetic cost on reproductive success?. Ecol. Entomol..

[50] Bosch J, Vicens N (2006). Relationship between body size, provisioning rate, longevity and reproductive success in females of the solitary bee Osmia cornuta. Behav. Ecol. Sociobiol..

[51] Wasielewski O, Giejdasz K, Wojciechowicz T, Skrzypski M (2011). Ovary growth and protein levels in ovary and fat body during adult-wintering period in the red mason bee, Osmia rufa. Apidologie.

[52] Lee KY, Lee KS, Yoon HJ, Jin BR (2015). Ovarian development and secretion of vitellogenin protein during the wintering period and after emergence in the hornfaced bee, Osmia cornifrons. J. Asia. Pac. Entomol..

[53] Sgolastra F (2018). Pesticide exposure assessment paradigm for solitary bees. Environ. Entomol..

[54] Sanchez-Bayo F, Goka K (2014). Pesticide residues and bees-a risk assessment. PLoS One.

[55] Cresswell JE (2012). Differential sensitivity of honey bees and bumble bees to a dietary insecticide (imidacloprid). Zoology.

[56] Thompson HM, Wilkins S, Harkin S, Milner S, Walters KFA (2015). Neonicotinoids and bumblebees (Bombus terrestris): effects on nectar consumption in individual workers. Pest Manag. Sci..

[57] Kessler SC (2015). Bees prefer foods containing neonicotinoid pesticides. Nature.

[58] Wu YY (2017). Sublethal effects of imidacloprid on targeting muscle and ribosomal protein related genes in the honey bee Apis mellifera L. Sci. Rep..

[59] Tosi S (2016). Effects of a neonicotinoid pesticide on thermoregulation of African honey bees (Apis mellifera scutellata). J. Insect Physiol..

[60] Potts R (2018). The effect of dietary neonicotinoid pesticides on non-flight thermogenesis in worker bumble bees (Bombus terrestris). J. Insect Physiol..

[61] Tosi S, Burgio G, Nieh JC (2017). A common neonicotinoid pesticide, thiamethoxam, impairs honey bee flight ability. Sci. Rep..

[62] Suchail S, Guez D, Belzunces LP (2001). Discrepancy between acute and chronic toxicity induced by imidacloprid and its metabolites in Apis mellifera. Environ. Toxicol. Chem..

[63] Tosi S, Nieh JC (2017). A common neonicotinoid pesticide, thiamethoxam, alters honey bee activity, motor functions, and movement to light. Sci. Rep..

[64] Williamson SM, Willis SJ, Wright GA (2014). Exposure to neonicotinoids influences the motor function of adult worker honeybees. Ecotoxicology.

[65] Crall JD (2018). Neonicotinoid exposure disrupts bumblebee nest behavior, social networks, and thermoregulation. Science.

[66] Feltham H, Park K, Goulson D (2014). Field realistic doses of pesticide imidacloprid reduce bumblebee pollen foraging efficiency. Ecotoxicology.

[67] Biddinger DJ, Rajotte EG (2015). Integrated pest and pollinator management-adding a new dimension to an accepted paradigm. Curr. Opin. Insect Sci..

[68] EFSA. European Food Safety Authority (2013). Guidance on the risk assessment of plant protection products on bees (Apis mellifera, Bombus spp. and solitary bees). EFSA J..

[69] Artz DR, Pitts-Singer TL (2015). Effects of fungicide and adjuvant sprays on nesting behavior in two managed solitary bees, Osmia lignaria and Megachile rotundata. PLoS One.

[70] Ladurner E, Bosch J, Kemp WP, Maini S (2005). Assessing delayed and acute toxicity of five formulated fungicides to Osmia lignaria Say and Apis mellifera. Apidologie.

[71] Ladurner E, Bosch J, Kemp WP, Maini S (2008). Foraging and nesting behavior of Osmia lignaria (Hymenoptera: Megachilidae) in the presence of fungicides: cage studies. J. Econ. Entomol..

[72] Scott-Dupree CD, Conroy L, Harris CR (2009). Impact of currently used or potentially useful insecticides for canola agroecosystems on Bombus impatiens (Hymenoptera: Apidae), Megachile rotundata (Hymentoptera: Megachilidae), and Osmia lignaria (Hymentoptera: Megachilidae). J. Econ. Entomol..

[73] Sgolastra F, Tosi S, Medrzycki P, Porrini C, Burgio G (2015). Toxicity of spirotetramat on solitary bee larvae, Osmia cornuta (Hymenoptera: Megachilidae), in laboratory conditions. J. Apic. Sci..

[74] Maienfisch P (2001). Chemistry and biology of thiamethoxam: A second generation neonicotinoid. Pest Manag. Sci..

[75] Manjon C (2018). Unravelling the molecular determinants of bee sensitivity to neonicotinoid insecticides. Curr. Biol..

[76] Higes M, Martín-Hernández R, Garrido-Bailón E, García-Palencia P, Meana A (2008). Detection of infective Nosema ceranae (Microsporidia) spores in corbicular pollen of forager honeybees. J. Invertebr. Pathol..

[77] Singh Rajwinder, Levitt Abby L., Rajotte Edwin G., Holmes Edward C., Ostiguy Nancy, vanEngelsdorp Dennis, Lipkin W. Ian, dePamphilis Claude W., Toth Amy L., Cox-Foster Diana L. (2010). RNA Viruses in Hymenopteran Pollinators: Evidence of Inter-Taxa Virus Transmission via Pollen and Potential Impact on Non-Apis Hymenopteran Species. PLoS ONE.

[78] Graystock P (2016). Hygienic food to reduce pathogen risk to bumblebees. J. Invertebr. Pathol..

[79] Sandrock C (2014). Sublethal neonicotinoid insecticide exposure reduces solitary bee reproductive success. Agric. For. Entomol..

[80] Bosch J, Vicens N (2002). Body size as an estimator of production costs in a solitary bee. Ecol. Entomol..

[81] Heinrich Bernd (1993). The Hot-Blooded Insects.

