# Author Correction: Chronic oral exposure to field-realistic pesticide combinations via pollen and nectar: effects on feeding and thermal performance in a solitary bee

**DOI:** 10.1038/s41598-021-95575-6

**Published:** 2021-08-09

**Authors:** Celeste Azpiazu, Jordi Bosch, Elisa Viñuela, Piotr Medrzycki, Dariusz Teper, Fabio Sgolastra

**Affiliations:** 1 Unidad de Protección de Cultivos, Escuela Técnica Superior de Ingeniería Agronómica, Alimentaria y de Biosistemas, Universidad Politécnica de Madrid (ETSIAAB-UPM), Av. Puerta de Hierro 2, 28040 Madrid, Spain; 2 CREAF, Universitat Autònoma de Barcelona, Cerdanyola del Vallès, 08193 Barcelona, Spain; 3 CREA-Consiglio per la Ricerca in Agricoltura e l’Analisi dell’Economia Agraria, Centro di Ricerca Agricoltura ed Ambiente, Via di Saliceto 80, 40128 Bologna, Italy; 4 Research Institute of Horticulture, Apiculture Division, 2 Kazmierska st., 24100 Puławy, Poland; 5 Dipartimento di Scienze e Tecnologie Agro-Alimentari, Alma Mater Studiorum Università di Bologna, viale Fanin 42, 40127 Bologna, Italy

Correction to: *Scientific Reports* 10.1038/s41598-019-50255-4, published online 24 September 2019

The original version of this Article contained errors.

In the Abstract,

“We measured pollen and syrup consumption, longevity, ovary maturation and thermogenesis. Pesticide intake was three orders of magnitude higher via syrup than pollen. At the tested concentrations, no synergistic effects emerged, and we found no effects on longevity and ovary maturation.”

now reads:

“We measured pollen and syrup consumption, longevity, ovary maturation and thermogenesis. Although bees consumed larger amounts of syrup than pollen, pesticide intake via syrup and pollen were similar. At the tested concentrations, no synergistic effects emerged, and we found no effects on longevity and ovary maturation.”

In the Results section, under subheading ‘Syrup and pollen consumption’,

“The total amounts of pesticide ingested via syrup and pollen by bees of each treatment throughout the entire exposure are reported in Table 2. In all cases, exposure via syrup was three orders of magnitude higher than exposure via pollen.”

now reads:

“The total amounts of pesticide ingested via syrup and pollen by bees of each treatment throughout the entire exposure are reported in Table 2.”

In the Discussion section,

“However, because solitary bee adults consume much greater amounts of nectar than pollen (ca. 93% of total food weight consumed by bees in our study was via syrup), the amount of active ingredient ingested per bee in our study was about three orders of magnitude higher via syrup than pollen.”

now reads:

“However, because solitary bee adults consume much greater amounts of nectar than pollen (ca. 93% of total food weight consumed by bees in our study was via syrup), the amounts of active ingredient ingested per bee in our study were similar via pollen and via syrup.”

And,

“As a result, the dose of imidacloprid (alone and in mixtures) ingested by *O. bicornis* females throughout their life-span was ca. 1.5 ng. This amount is 10–20 times lower than the acute oral LD_50_ reported in honey bees (13 ng bee^−1^ ^54^) and bumblebees (27 ng bee^−1^ ^54^). For the same reason, the amounts of acetamiprid and/or myclobutanil ingested by bees in A + I, I + M and A + I + M were also reduced by 80% when compared to treatments containing acetamiprid and myclobutanil but not imidacloprid (Table 2). Feeding suppression following exposure to this neonicotinoid has also been reported in *A. mellifera*^29^ and *Bombus terrestris* L.^47,55,56^. Because bees cannot taste neonicotinoids^57^, feeding suppression is likely to be due to the toxicity of the neonicotinoid rather than repellence. Kessler et al.^57^ found that honey bees and bumblebees preferred syrup containing imidacloprid to control solutions, even though ingestion of this compound caused them to eat less syrup overall. We found feeding suppression in *O. bicornis* exposed to imidacloprid at doses as low as 1.27–1.64 ng bee^−1^.”

now reads:

“As a result, the dose of imidacloprid (alone and in mixtures) ingested by *O. bicornis* females throughout their life-span was ca. 4–9 ng. This amount is 1.4–6.8 times lower than the acute oral LD_50_ reported in honey bees (13 ng bee^−1^ ^54^) and bumblebees (27 ng bee^−1^ ^54^). For the same reason, the amounts of acetamiprid and/or myclobutanil ingested by bees in A + I, I + M and A + I + M were also reduced by 80% when compared to treatments containing acetamiprid and myclobutanil but not imidacloprid (Table 2). Feeding suppression following exposure to this neonicotinoid has also been reported in *A. mellifera*^29^ and *Bombus terrestris* L.^47,55,56^. Because bees cannot taste neonicotinoids^57^, feeding suppression is likely to be due to the toxicity of the neonicotinoid rather than repellence. Kessler et al.^57^ found that honey bees and bumblebees preferred syrup containing imidacloprid to control solutions, even though ingestion of this compound caused them to eat less syrup overall. We found feeding suppression in *O. bicornis* exposed to imidacloprid at doses as low as 0.2–0.5 ng bee^−1^ day^−1^.”

In addition, Table 2 contained errors. The values for ‘Period 1’, ‘Period 2’ and ‘Total’ were incorrectly given.

The original Table 2 and accompanying legend appear below.

**Table 2:** Body weight and amount of active ingredient ingested via syrup and pollen in *O. bicornis* females exposed to various pesticide combinations (treatments) throughout their adult life span (chronic exposure).

Treatment	n bees	Body weight (mean ± SE mg)	Acetamiprid (mean ng bee^−1^)				Imidacloprid (mean ng bee^−1^)				Myclobutanil (mean ng bee^−1^)
			Syrup	Pollen		Total	Syrup	Pollen		Total	Syrup
				Period 1	Period 2			Period 1	Period 2		
A	20	70.67 ± 1.87	2.88	0.007	0.0003	2.8902					
I	16	68.93 ± 1.58					1.63	0.008	0.0006	1.6375	
M	17	71.09 ± 2.03									3.42
A + I	13	69.08 ± 2.85	0.58	0.005	0.00004	0.5883	1.40	0.004	0.00003	1.4002	
A + M	16	72.31 ± 1.72	3.34	0.01	0.0003	3.3520					2.91
I + M	12	71.50 ± 2.37					1.59	0.008	0.0003	1.5999	0.58
A + I + M	14	66.72 ± 2.16	0.53	0.007	0.0002	0.5356	1.27	0.005	0.0001	1.2707	0.46
CONTROL	13	68.19 ± 3.01									

A: acetamiprid, I: imidacloprid, M: myclobutanil. Period 1: first week; Period 2: remainder of the bioassay.

The original Article has been corrected.

