# Supplementary material for: Chronic oral exposure to field-realistic pesticide combinations via pollen and nectar: effects on feeding and thermal performance in a solitary bee
Source: Sci Rep. 2019 Sep 24;9:13770. doi: 10.1038/s41598-019-50255-4 (PMC6760219; doi:10.1038/s41598-019-50255-4)
Supplement: Supplementary file 1 — Suplementary Information [file 41598_2019_50255_MOESM1_ESM.pdf]

SUPPLEMENTARY INFORMATION FOR:

Chronic oral exposure to field-realistic pesticide combinations via pollen and nectar: effects on feeding and thermal performance in a solitary bee

Celeste Azpiazu[1,*], Jordi Bosch[2], Elisa Viñuela[1], Piotr Medrycki[3], Dariusz Teper[4], Fabio Sgolastra[5]

[1]Unidad de Protección de Cultivos, Escuela Técnica Superior de Ingeniería Agronómica, Alimentaria y de Biosistemas, Universidad Politécnica de Madrid (ETSIAAB-UPM), Av. Puerta de Hierro 2, 28040 Madrid, Spain

[2]CREAF, Universitat Autònoma de Barcelona, Cerdanyola del Vallès, 08193 Barcelona, Spain

[3]CREA-Consiglio per la Ricerca in Agricoltura e l'Analisi dell'Economia Agraria, Centro di Ricerca Agricoltura ed Ambiente, Via di Saliceto 80, 40128 Bologna, Italy

[4]Research Institute of Horticulture, Apiculture Division, 2 Kazmierska st., 24100 Puławy, Poland

[5]Dipartimento di Scienze e Tecnologie Agro-Alimentari, Alma Mater Studiorum Università di Bologna, viale Fanin 42, 40127 Bologna, Italy

* Corresponding author.
E-mail address: celeste.azpiazu@upm.es

CONTENT

- Supplementary Information for Methods section
- Video caption S1

Supplementary Information for Methods section

- Determination of pesticide levels in pollen and nectar of melon flowers

1. Melon flower sampling: nectar and pollen collection

Pollen and nectar collection were carried out in the Crop Protection Unit, School of Agricultural, Food and Biosystem Engineering (UPM), Madrid, Spain. During July 2017 we collected melon flowers in five conventional fields on the basin of the Tajo River near Madrid (Spain). Nectar was extracted from 30-70 flowers using microcapillaries (5µl Blaubrand® intraMARK) to obtain an amount of 50µl per sample (n=3 per field), and then stored at -80°C. Hundreds of additional flowers were frozen at -80°C for later pollen collection. In October, these flowers were dried in an incubator at 37 °C for 24 h to facilitate pollen removal [1]. Anthers were then collected and the pollen was extracted using a sieve of 150µm pore size (melon pollen grain Ø = 50-100µm [2]). We used 140-250 flowers to obtain approximately 0.1g of pollen per sample (n=3 per field).

2. Multi-residue analyses of pesticides

The multi-residual analyses were carried out in the Laboratorio Químico Microbiológico de Sevilla an official accredited analytical testing company (www.lqmsa.com). High performance liquid chromatography with quantification and confirmation by triple-quadrupole mass spectrometer detector (HPLC-QQQ) and gas chromatography with triple-quadrupole mass spectrometer detector (GC-QQQ) were used to target more than 200 compounds. For the 3 pesticides used in our trials, imidacloprid and acetamiprid were analyzed by HPLC-QQQ and myclobutanil by GC-QQQ. The recoveries were over the detection limit (LOD) of 3ng/g for each analyte.

2.1. Pollen and nectar preparation

Pollen preparation was performed using a modified version of the QuEChERS methodology described by David et al. [3]. Pollen samples (100-200mg) were weighed and introduced in 15-ml Eppendorf Tubes with a ceramic microbead Two ml of water were added to each sample to form an emulsion and extracted by adding 2ml of acetonitrile ($CH3CN$, Baker 9012 “HPLC Analyzed), mixing in Agytax® SR2 for 2 minutes and sonicating for 10 minutes. Then, 1g of magnesium sulphate/sodium acetate

mix (4:1) (QuEChERS Salts, Agilent 5982-0650) was added followed by immediate shaking (Agytax® SR2) for 2 min and sonication without heating for 10 min. The supernatant was transferred into a 4-ml polypropylene tube with a ceramic microbead and Agilent QuEChERS dispersive 5982-5056 was added to match the weight of the sample and shaken for 2 min (Agytax® SR2) and centrifugated (3000 rpm for 5 min). The supernatant was measured and transferred to a centrifuge tube and dried with high purity N_2 stream. The extract was reconstituted with 100µL of CH3CN.

Nectar preparation followed a similar procedure. To calculated nectar weight, nectar samples (ca. 50µL) were inserted in pre-weighed Eppendorf tubes. The volume of water added was adjusted to 1mL.

2.2. Instruments

HPLC-QQQ was performed with an Agilent Technologies 1200 HPLC consisting of a binary pump G1312A, an autosampler, a vacuum degasser and a thermo column compartment (G1316A). The analytical column for the separation of the analytes was an Agilent Technologies Poroshell 120 C18 (10cm × 2.1mm × 2.7µm) with adequate guard column maintained at 40°C. The separation of the analytes was performed by applying a gradient of components A (CH3CN with 0.1% formic acid) and B (2mM ammonium formiate in water with 0.1% formic acid) with a flow rate of 0.6 $mL \cdot min^{-1}$. The injection volume was 5µL. The gradients started with 20% of component A and 80% of component B for 1 min and then the component A was increased to 100% within 10 min. After for 4 min, the component A was decreased to 20% and the component B increased to 80% within 1 min, followed by equilibration for 5 min. Total run time was 22 min.

The Agilent Technologies 6410 mass spectrometer working in dynamic multi reaction monitoring (MRM) mode is equipped with an electrospray ionization (ESI) interface for the introduction of solvated samples. Triple Quadrupole Mass Spectrometer was used for the detection and quantification of pesticides. Capillary positive voltage was 3000V. Nitrogen was the nebulising gas with a flow rate of $9L \cdot min^{-1}$ and temperature 345°C. Nebulizer gas pressure was set at 40 psi.

To perform the GC-QQQ, we used a GC Agilent Technologies GC7890A with an automatic liquid autosampler and a split-splitless injector. A DB-5 (5%Phenyl 95%

Methylpolysiloxane) column Agilent 19091S-433 (30m × 0, 25mm × 0.25 µm) was used. A sample volume of 1 µL was injected into the GC in splitless mode at an injector temperature of 250°C. The oven temperature program was as follows: initial temperature 70 °C (held for 2 min) increased by 25°C/min to 150°C; increased by 3°C /min to 200°C (held for 1 min); increased by 8°C/min to 280°C (held for 10 min). High Purity Nitrogen gas was used as collision gas with a flow rate of 1.5mL/min and Helium at 1.35mL/min for quenching effect into the collision cell (octopole). The concentration of the calibration standards were 2 to 100µg/L

References

1. Botías, C. *et al.* Neonicotinoid residues in wildflowers, a potential route of chronic exposure for bees. *Environ. Sci. Technol.* **49,** 12731–12740 (2015).

2. PalDat. PalDat- Palynologgical Database. (2017). at <https://www.paldat.org/>

3. David, A., Botías, C., Abdul-Sada, A., Goulson, D. & Hill, E. M. Sensitive determination of mixtures of neonicotinoid and fungicide residues in pollen and single bumblebees using a scaled down QuEChERS method for exposure assessment. *Anal. Bioanal. Chem.* **407,** 8151–8162 (2015).

Video S1: Recordings of *O. bicornis* females after 11 days of chronic exposure to imidacloprid and acetamiprid, respectively.
